# Supplementary material for: Erythro–Magneto–HA–Virosome: A Bio-Inspired Drug Delivery System for Active Targeting of Drugs in the Lungs
Source: Int J Mol Sci. 2022 Aug 31;23(17):9893. doi: 10.3390/ijms23179893 (PMC9455992; doi:10.3390/ijms23179893)
Supplement: Supplementary file 1 [file ijms-23-09893-s001.zip › ijms-1792598-supplementary.pdf]

International Journal of *Molecular Sciences*

MDPI

Supplementary Figures

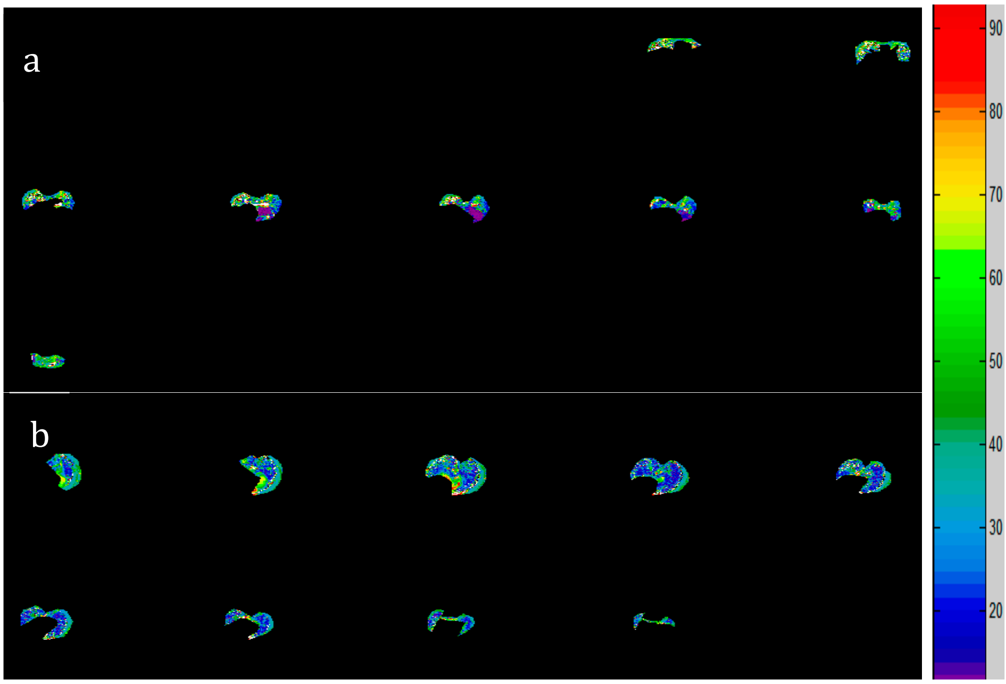

Figure S1: NMR Description of the T2 signal mapping for the entire frontal to caudal scanning of the mouse lung. (**a**) T2 was recorded after EMHV administration without application of the magnetic field; (**b**) T2 was recorded after EMHV administration and application of the magnetic field for 30 min. These images were used to create the 3D reconstruction of the images in figure 5c .

Naive animal: no EMHV and no magnetic field

EMHV and no magnetic field@ lung

EMHV and magnetic field application (30 min) @ lung

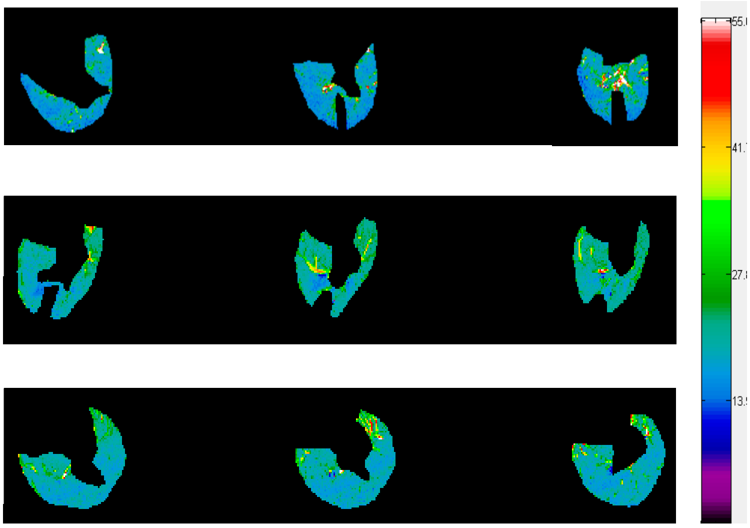

Figure S2: NMR Description of the T2 signal mapping of selected frontal to caudal sections of the mouse liver. (**a**) images of T2 map in a naive mouse. (**b**) after EMHV administration without application of the magnetic field; (**c**) after EMHV administration and application of the magnetic field for 30 min @ lung. In a, b and c, the T2 signal maps do not show any evident perturbation showing that no preferential accumulation was detected in any of the cases due to EMHV (**b**,**c**) when compared to naïve (a, control). These results demonstrate that circulating EMHV do not accumulate in the liver with or without application of magnetic field

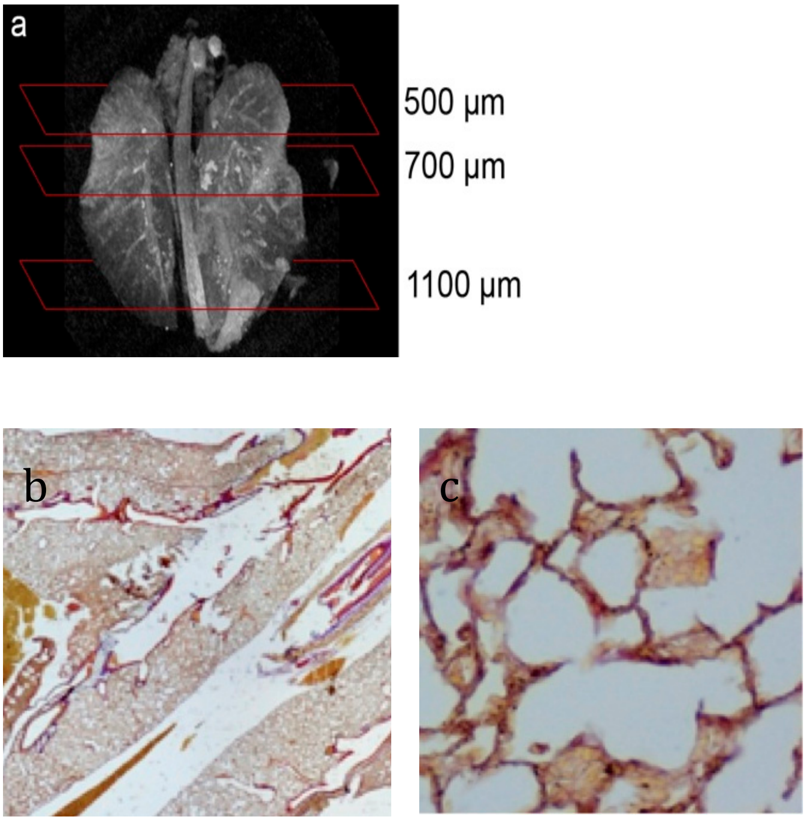

Figure S3: (**a**) CT imaging of paraformaldehyde fixed lungs embedded in paraffin to study thee presence of EMHV accumulation. Immuno-hystochemical investigation (**b**,**c**) shows no enrichment of EMHV at 1100 by Perl's Prussian Blue reactivity due to Iron particles. (**b**) 20× and (**c**) 40× magnification
